# Evolution of Translational Bioinformatics: lessons learned from TBC 2016

**DOI:** 10.1186/s12920-017-0262-5

**Published:** 2017-05-24

**Authors:** Kye Hwa Lee, Ju Han Kim

**Affiliations:** 1 Biomedical Informatics, Seoul National University Hospital, 28 Yongon-dong, Chongno-gu, Seoul, 110799 Korea; 2 Div. of Biomedical Informatics, Seoul National University College of Medicine, 28 Yongon-dong, Chongno-gu, Seoul, 110799 Korea

## Introduction

Translational bioinformatics (TBI) is a relatively young discipline that spans a wide spectrum from data to diagnostics and therapeutics. TBI involves applying novel methods to the storage, analysis, and interpretation of a massive volume of omics data, and it bridges the gap between bench research and real-world application to human health. The Translational Bioinformatics Conference (TBC) series has aimed to highlight the multidisciplinary nature of TBI, and it provides an opportunity to bring researchers together to exchange ideas between biology, informatics, technology, and clinical fields worldwide.

Since its inauguration in 2011, the TBC series has grown into one of the most successful international multidisciplinary conference series. The first 3 years of the conferences were hosted by Korean scientific societies, while TBC 2014 was hosted by the Chinese Academy of Sciences in Huiquan, China, and TBC 2015 was hosted by the Japanese Association of Medical Informatics in Tokyo, Japan. TBC 2016 was hosted again in Seoul, Korea, and TBC 2017 will be held at the Meyer and Rene Luskin Center in Los Angeles, CA, United States. There has been a total of 255 research presentations in the TBC series, involving 18 participating countries: Australia, Canada, China, Colombia, Denmark, India, Israel, Japan, Korea, Luxembourg, Poland, Saudi Arabia, South Africa, Spain, Sweden, Taiwan, the United Kingdom, and the United States. Seventy-six papers have been published in high-quality journals, including the Journal of American Medical Informatics, BMC Medical Genomics, and BMC Medical Informatics, and a further 15 are in press.

The studies reported on at TBC 2016 mainly focused on inferences of new knowledge by the flexible application of informatics methodology to various dimensions of omics data, and suggested ways to apply this knowledge to clinical practice. The success of the TBC series indicates that the explosion of high-throughput data is changing the paradigm of medicine to being proactive, preventive, and precise, by creating new knowledge through interdisciplinary research using both traditional and evolutionary informatics methods.

## Genomics applications for precision medicine

There is a need for new research methodologies for interpreting genomics data and applying them to clinical practice. Research into methods for determining the contribution of rare mutations to diseases has recently become one of the most important areas of genome research. Kim et al. (Indiana University, USA) propose a novel biological knowledge-driven binning approach (called Bin-KAT) to identify trait- and disease-associated rare variants [1]. Those authors applied the method to whole-genome sequencing data to identify rare variants associated with a neuroimaging endophenotype related to late-onset Alzheimer’s disease (LOAD), and identified 16 functional exonic rare variants in *FANCC* that were significantly associated with the thickness of the entorhinal cortex.

Studies of diseases involving the brain metabolism (e.g., Alzheimer’s disease) that have utilized imaging tests or other biomarkers as surrogate markers and combined them with genetic data have recently achieved good results. Nho et al. (Indiana University, USA) found that rare variants in the region surrounding *APOE* on chromosome 19 were significantly associated with LOAD-related CSF antibody and neuroimaging biomarkers after adjusting for the *APOE* genotype [2]. Combining genomic data with other quantitative biomarkers yields a better understanding of the underlying mechanisms of neural degeneration and other brain diseases and also the potential for additional potential diagnostic and therapeutic targets.

Lee et al. (Ajou University, Korea) propose a novel method that incorporates subtype information to better explain the variability in gene expression based on the methylation profile [3]. They address the issue of infeasibility of separately estimating association patterns between gene expressions and DNA methylation features by employing a kernel-weighted lasso model that can incorporate information from samples in different subtypes while allowing subtype-specific estimations. They demonstrate that this model can be used to discover subtype-sensitive genes that a plain lasso framework can not detect. Wang et al. (Ajou University, Korea) constructed advanced gene–gene interaction networks from SNP-based epistasis networks to identify multiple genetic risk factors associated with the disease from a genome-wide association study data set [4]. They report that the genes identified in the integrative network driven by the SNP data for aspirin-exacerbated respiratory disease were successfully validated in DisGeNET data and provide meaningful information about the biological ontology.

## Inferring clinical significance through integrating heterogeneous omics data

The rapid increase in high-dimensional multilayer omics data including transcriptomics, proteomics, and metabolomics, as well as in genomics data has led to a shift in the medical paradigm from existing paradigms that rely on signs and symptoms to define diseases to a new paradigm that redefines disease based on the underlying molecular pathway. Shivakumar et al. (Geisinger Health System, USA) propose an integrative framework for identifying epigenetic interactions between methylation and miRNA associated with transcriptomic changes [5]. Their approach is valuable since a mechanistic role of synergistic interactions between DNA methylation and miRNA as epigenetic regulators on transcriptomic changes and its association with clinical outcomes such as survival have remained largely unexplored in cancer. Those authors identified epigenetic interactions that are associated with survival as potential prognostic markers in bladder cancer by exploration of TCGA bladder cancer data.

Omics data present a challenge to traditional statistical methods in obtaining meaningful results from the excessive data in different dimensions. Kim et al. (Ajou University, Korea) integrated multilayered omics data to represent novel knowledge and applied it to a disease co-occurrence prediction problem on a two-layer network consisting of symptom and disease layers [6]. Their results showed the relevance of using symptoms for predicting disease co-occurrence, presenting statistical evidence for the average number of shared symptoms being higher for co-occurring diseases than for non-co-occurring diseases. Predicting disease co-occurrence prediction is important for practical treatments and prevention methods.

One of the powerful achievements of TBI methods is that they enable N-of-1 studies and the consequent accumulation of a considerable amount of new knowledge from them. Li et al. (The University of Arizona, USA) propose advanced methods for analyzing personal transcriptomes derived from a pair of samples of a single patient [7]. To complement previously published methods [8], those authors developed the N-of-1-pathways MixEnrich mixture model, which combined with a gene set enrichment test could be used to uncover bidirectional and concordantly dysregulated pathways in individual patients. Bidirectional and concordant dysregulated pathways uncovered by MixEnrich in each patient largely overlapped with the quasi-gold standard, in contrast to other single-subject and cohort-based transcriptome analyses confirmed in both the simulation study and data on head and neck squamous cell carcinoma.

Considerable challenges in dementia are the inability to prevent the disease at the time of a correct diagnosis and the irreversibility of disease progression. Bang at el. (Ajou University, Korea) suggest a four-step model consisting of proposer, predictor, descriptor and visualization modules, which are used to construct a prediction model that integrates various dementia diagnosis techniques using a machine-learning technique and also provides easy-to-understand results and visualizations [9]. Those authors considered not only the predictive performance of the model but also the detailed interpretation of results, which is needed for physicians to be able to usefully interpret the prediction results and hence increases the clinical utility of the model.

## Systems medicine and network modeling

The functions of the biological basic units of living organisms—such as genomes, proteins, tissues, and organs—react closely to changes in the external environment and maintain life by constantly adapting. One of the key areas of TBI is system medicine, which derives an understanding of diseases and life phenomena by studying complex interactions at each unit and level from the point of view of the complete biological system.

Park et al. (Ajou University, Korea) propose a methodology for implementing drug repositioning by maximizing the effectiveness in terms of both time and cost [10]. The main assumption in that paper is that similar diseases can be treated using similar drugs, called network mirroring, and those authors applied this to dementia. The drugs selected using network mirroring coincide with the existing drugs being used, while their approach also discovered drugs with great potential for repositioning and verified the drugs based on the clinical literature.

The importance of exploring noncoding regions of the genome is increasing given that many genetic diseases are caused by mutations in these areas. Herman-Izycka et al. (University of Warsaw, Poland) describe a random-forest-based machine learning approach that demonstrates that enhancer sequences can only be active under a closely defined set of circumstances, and which defines a set of features that are crucial for predicting the activity [11].

There has been considerable progress in the development of a disease network for obtaining new knowledge and improving the prognosis and predicting the treatment outcome of disease. Lee et al. (Ajou University, Korea) constructed a causal disease network using a biomedical literature text-mining method to complement the existing disease network, without needing to consider the causality or posterior relationship of the disease [12]. In order to predict the causality of a disease, they proposed two types of term for quantifying the strength of causal relationships between diseases from the biomedical literature: lexicon- and frequency-based terms.

## Methods for advanced translation

The predominant predictions in TBI do not only influence the decisions made by physicians but also the motivation and engagement of patients, which can promote their participation in participatory medicine. Nam et al. (Ajou University, Korea) developed a new model for the hearing gain to be expected after fitting hearing aids based on variables that can affect the outcomes [13], and proposed a novel neural-network algorithm for implementing the model.

A life log is another tool for encouraging patient-centered medicine combined with bioinformatics analysis. The invention of fitness trackers has made it possible to continuously monitor a user’s biometric data, such as the heart rate, number of footsteps, and amount of calories used. The paper written by Kim et al. (Korea Advanced Institute of Science and Technology, Korea) defines and investigates the notion of a user’s “activeness,” and shows that it is possible to forecast the long-term activeness of a user [14]. Such information can be utilized by a health-related application to proactively recommend suitable events or services to individual users.

Carson et al. (University of Illinois at Chicago, USA) developed a method to measure disease similarity that incorporates the uniqueness of shared genes using OMIM and Disease Ontology annotation [15]. The authors used the Disease-Ontology-based matrix to identify several interesting connections between cancer and other diseases and conditions (e.g., malaria), along with studies to support the findings.

We have shown that creative interactions involving various biomedical informatics research methodologies have been applied freely at TBC 2016 to a wide range of data—from biological data such as genomics or transcriptomics to life-log and literature data—to obtain new knowledge that can be translated to medical applications. I congratulate the speakers and authors at this conference who are active evolving translational medicine into innovative health care. Many health topics are increasingly falling within the scope of TBI, including rare and complex human diseases, cancer, biomarkers, pharmacogenomics, drug repositioning, and clinical decision support systems. The evolving clinical decision support tools and the accumulation of translational knowledge are together sharply decreasing the distance from the bench to the bedside.
